# Relative Efficacy of Immunomodulatory Monotherapies for Psoriasis of the Scalp: A Network Meta‐Analysis Study

**DOI:** 10.1111/jocd.70662

**Published:** 2026-01-08

**Authors:** Aditya K. Gupta, Mary A. Bamimore, Tong Wang, Vincent Piguet, Mesbah Talukder

**Affiliations:** ^1^ Mediprobe Research Inc. London Ontario Canada; ^2^ Division of Dermatology, Temerty Faculty of Medicine University of Toronto Toronto Ontario Canada; ^3^ Division of Dermatology Women's College Hospital Toronto Ontario Canada; ^4^ School of Pharmacy BRAC University Dhaka Bangladesh

## Abstract

**Background:**

Recently, the literature has expanded with peer‐reviewed studies on immunomodulatory agents' efficacy on scalp psoriasis—which, in turn, widened knowledge gaps regarding these agents' relative effectiveness. We determined the relative efficacy of immunomodulatory monotherapies for scalp psoriasis.

**Methods:**

We ran Bayesian network meta‐analyses (NMAs) using outcomes related to Psoriasis Scalp Severity Index (PSSI) and scalp‐specific Physician's Global Assessment of clear (0) or almost clear (1) (Sc‐PGA 0/1).

**Results:**

We estimated the relative efficacy of 22 interventions (including placebo), and analyzed 9 outcomes, namely: proportion of participants who attained Sc‐PGA 0/1, proportion of participants who achieved 100% improvement in PSSI (PSSI‐100), and proportion of participants who achieved 90% improvement in PSSI (PSSI‐90) at 8, 12, and 16 weeks.

**Conclusions:**

We are the first to provide comparative evidence on the efficacy of newly investigated agents such as deucravacitinib, tildrakizumab, roflumilast and icotrokinra. In general, the IL‐17 inhibitors (bimekizumab, ixekizumab, secukinumab, brodalumab) and IL‐23 inhibitors (icotrokinra, guselkumab, tildrakizumab) were effective depending upon the outcome and time‐point being considered. At 16 weeks, for PSSI‐100, ixekizumab 150 mg at weeks 0, 2, 4, 8, and 12 ranked highest; at 16 weeks, for Sc‐PGA 0/1 bimekizumab 320 mg every 4 weeks ranked highest; at 8 weeks, for PSSI‐100 ixekizumab 80 mg every 2 weeks ranked highest; at 8 weeks, for Sc‐PGA 0/1 secukinumab 300 mg at weeks 1, 2, 3 and then every 4 weeks ranked highest. Small‐molecule therapies (apremilast, deucravacitinib, roflumilast) improved scalp psoriasis modestly. Our work would guide the design of future studies and clinical decision‐making.

## 1 Introduction

In approximately 80% of patients with plaque psoriasis, there is scalp involvement [1], and psoriasis of cranial skin can be challenging to treat because of the scalp's occupancy of hair. Scalp psoriasis has psychopathological consequences [2] because the condition is strongly correlated with diagnoses of anxiety and depression—each of which negatively impacts quality of life. Various therapeutic agents have been used to treat scalp psoriasis; among the most recent ones are the immunomodulatory agents such as monoclonal antibodies, interleukin‐17A antagonists, phosphodiesterase 4 (PDE4) inhibitors, and tyrosine kinase 2 inhibitors [3].

To our knowledge, the first network meta‐analysis (NMA) study that evaluated the management of scalp psoriasis using biologics was by Kaur et al., who compared the relative efficacy of immunomodulatory agents [4]. The efficacy of several other immunomodulatory monotherapies has been reported since publication of Kaur et al.'s work; the current study has investigated the comparative effectiveness of immunomodulatory treatments using data from the updated evidence base.

## 2 Methods

The conduct of our work was in accordance with the *Preferred Reporting Items for Systematic reviews and Meta‐Analyses* (PRISMA) guidelines for NMAs [5]. The protocol for our work was registered in the Open Science Framework (OSF) platform (registration link: https://osf.io/9s8ng).

### 2.1 Eligibility Criteria

As per the patient, intervention, comparator, outcome (PICO) framework, eligible trials were randomized, published in English, had an arm investigating the efficacy of monotherapy with an immunomodulatory agent on scalp psoriasis, and used outcome measures related to scalp‐specific Physician's Global Assessment (Sc‐PGA) and Psoriasis Scalp Severity Index (PSSI).

### 2.2 Search

We systematically searched the peer‐reviewed literature through PubMed and Scopus from January 1, 2015 onwards to identify eligible trials for our NMAs. Deduplication was conducted using the “Deduplicator” module of the Systematic Review Accelerator (SRA) web application [6]. The screening of titles and abstracts was done independently by two authors (TW and MAB)—and so too was the screening of full texts; any disagreements through the screening stages were resolved through discussion with a third author (AKG). The Rayyan software [7] was our tool of choice for assisting with the screening stages. Extracted data were organized in spreadsheets; we collected details including data on study characteristics, interventions’ regimen, and efficacy data.

### 2.3 Network

An NMA was conducted for each outcome measure. Each outcome was represented by a network, a graph of nodes and edges—where each node represents a comparator, and an edge corresponds to the comparison of two interventions that were investigated in a head‐to‐head trial. The geometry of a network determines whether node‐splitting analyses for inconsistency are feasible—and such an analysis is performed to determine whether direct evidence is not inconsistent with indirect evidence [8]. Nodes were analyzed at the level of dosage.

### 2.4 Network Meta‐Analyses

After the data extraction stage, we identified sufficient data to conduct NMAs for three specific outcome measures at 3 distinct time points; so, the 9 networks corresponded to: proportion of participants who achieved 100% improvement in PSSI (PSSI‐100), proportion of participants who achieved 90% improvement in PSSI (PSSI‐90), and proportion of participants who attained a Sc‐PGA or 0 (clear) or 1 (almost clear) (Sc‐PGA 0/1) at 8, 12, and 16 weeks. Each NMA was conducted under a Bayesian fixed‐effects model with uniform priors using the *multinma* R package [9] in RStudio (R version 4.3.2) [10].

Each NMA estimated the comparator's surface under the cumulative ranking curve (SUCRA), a metric that ranks a comparator's efficacy in relation to other comparators—and whose value ranges from 0 to 1 (or 0% to 100%). Each comparator's SUCRA value is estimated from rank probabilities, following the equation below [11]:

$${\text{SUCRA}}_{k}=\frac{\sum_{c=1}^{b-1}{\mathrm{Cum}}_{k,c}}{b-1}$$

where *b* and *k* represent the total number of therapies and specific treatment, respectively; the “*c* = 1” term represents the highest ranked comparator. Comparators SUCRA across all outcome measures were presented in a kilim plot [12].

Each NMA also estimated the comparators’ pairwise relative effects. Each pairwise relative effect corresponded to the mean difference (MD) between the log odds; a 95% credible interval (CI) was estimated for each mean difference. Placebo and vehicle arms were amalgamated into one node (i.e., “control”). Relative effects were presented in league tables.

### 2.5 Sensitivity Analyses

For each network, we additionally conducted NMAs that separately adjusted for variation in age and sex as our sensitivity analyses; in other words, we conducted network meta‐regressions—and the purpose thereof was to determine if relative efficacy was consistent across the base model and when adjusted for potential confounders such as age and sex. Hence, 9 NMAs corresponded to base analyses and 18 NMAs corresponded to sensitivity analyses [7]. Cochrane Collaboration's Risk of Bias (RoB) tool [13] was used to qualitatively assess each eligible study's evidence quality.

## 3 Results

**FIGURE 1:**
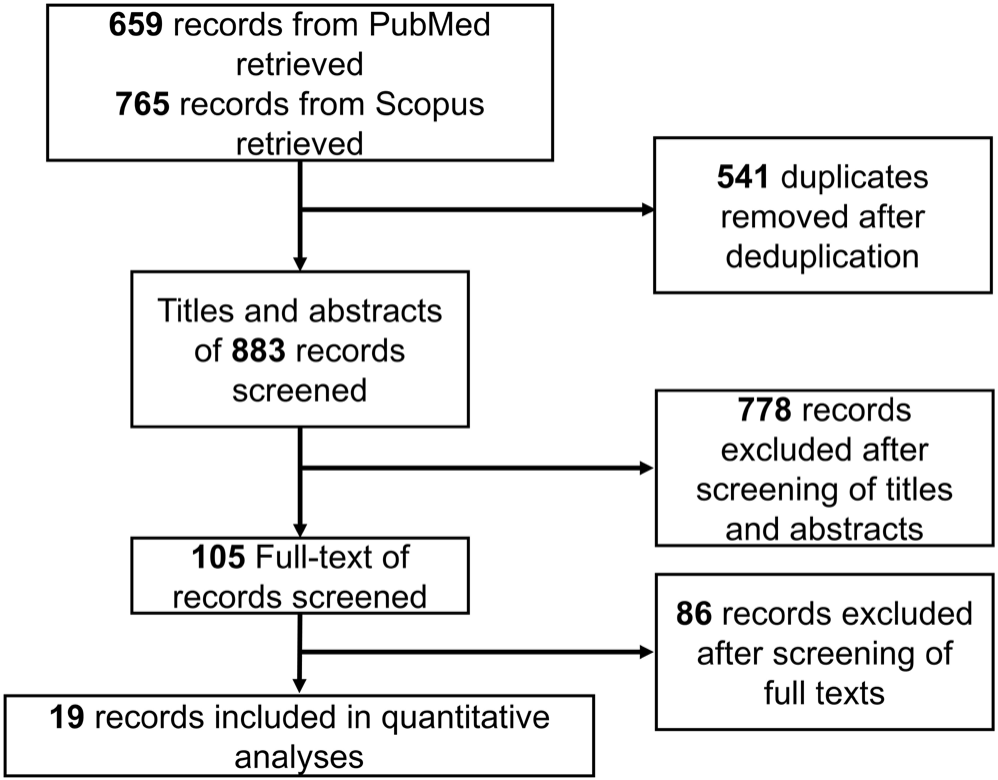
Identification of studies. This flowchart pictorially summarizes the search process involved in identifying studies whose data were eligible for our network meta‐analyses.

We identified 19 published studies [14, 15, 16, 17, 18, 19, 20, 21, 22, 23, 24, 25, 26, 27, 28, 29, 30, 31, 32] whose data were used to conduct all quantitative analyses, and across which 21 active comparators were identified (Figure 1, Table 1), namely, adalimumab 80 mg at week 0, 1 (subcutaneous), apremilast 30 mg twice daily (oral), bimekizumab 320 mg every 4 weeks (subcutaneous), deucravacitinib 6 mg once daily (oral), guselkumab 100 mg at week 0, 4, 12 (subcutaneous), tildrakizumab 100 mg at week 0, 4 (subcutaneous), roflumilast foam 0.3% once daily (topical), secukinumab 300 mg at week 1, 2, 3, then every 4 weeks (subcutaneous), ixekizumab 80 mg every 2 weeks (subcutaneous), ixekizumab 80 mg every 4 weeks (subcutaneous), ixekizumab 10 mg at week 0, 2, 4, 8, 12, 16 (subcutaneous), ixekizumab 25 mg at week 0, 2, 4, 8, 12, 16 (subcutaneous), ixekizumab 75 mg at week 0, 2, 4, 8, 12, 16 (subcutaneous), ixekizumab 150 mg at week 0, 2, 4, 8, 12, 16 (subcutaneous), brodalumab 210 mg every 2 weeks, icotrokinra 200 mg once daily (oral), icotrokinra 100 mg twice daily (oral), icotrokinra 100 mg once daily (oral), icotrokinra 50 mg once daily (oral), icotrokinra 25 mg twice daily (oral), and icotrokinra 25 mg once daily (oral). The control node amalgamated data from the vehicle and placebo arms.

**TABLE 1:** Summary of study‐level characteristics of included studies.

References	Arms	*N*	Age (mean)	Sex (males, %)
Gooderham et al. (2025) [17]	Roflumilast foam 0.3% once daily (topical)	281	48.6	45.9
	Control	151	45	39.7
Ferris et al. (2025) [32]	Icotrokinra 100 mg twice daily (oral)	36	NA	
	Icotrokinra 100 mg once daily (oral)	40		
	Icotrokinra 50 mg once daily (oral)	40		
	Icotrokinra 25 mg twice daily (oral)	32		
	Icotrokinra 25 mg once daily (oral)	37		
	Control	35		
Gold et al. (2025) [15] (ICONIC‐ADVANCE 1)	Deucravacitinib 6 mg once daily (oral)	307	46.3	65
	Icotrokinra 200 mg once daily (oral)	311	47.1	72
	Control	156	46.9	67
Gold et al. (2025) [15] (ICONIC‐ADVANCE 2)	Deucravacitinib 6 mg once daily (oral)	327	45.6	68
	Icotrokinra 200 mg once daily (oral)	322	45.9	68
	Control	82	48.4	67
Li et al. (2025) [18]	Ixekizumab 80 mg every 2 weeks (subcutaneous)	172	39.3	80.2
	Ixekizumab 80 mg every 4 weeks (subcutaneous)	170	41.1	74.1
	Control	84	41.2	72.6
McMichael et al. (2025) [19]	Guselkumab 100 mg at week 0, 4, 12 (subcutaneous)	76	42.9	52.6
	Control	26	41.1	69.2
Sofen et al. (2025) [22]	Tildrakizumab 100 mg at week 0, 4 (subcutaneous)	88	44.2	66.3
	Control	79	45.4	53.7
Gold et al. (2025) [15]	Guselkumab 100 mg at week 0, 4, 12 (subcutaneous)	225	47	51.6
	Control	113	44.5	50.4
Blauvelt et al. (2024) [25]	Deucravacitinib 6 mg once daily (oral)	514	44.5	64.6
	Apremilast 30 mg twice daily (oral)	276	45.6	59.8
	Control	294	46.6	66.3
Gebauer et al. (2024) [27]	Tildrakizumab 100 mg at week 0, 4 (subcutaneous)	89	44.2	66.3
	Control	82	45.4	53.7
NCT03370133	Bimekizumab 320 mg every 4 weeks (subcutaneous)	321	45.2	71.3
	Control	83	49.7	72.3
Jo et al. (2023) [21]	Guselkumab 100 mg at week 0, 4, 12 (subcutaneous)	94	41.2	77.7
	Adalimumab 80 mg at week 0, 1 (subcutaneous)	60	38.1	83.3
	Control	45	42.6	68.9
Kircik et al. (2023) [29]	Roflumilast foam 0.3% once daily (topical)	200	45.2	48
	Control	104	45	45.2
Orbai et al. (2023) [20]	Guselkumab 100 mg at week 0, 4, 12 (subcutaneous)	153		
	Adalimumab 80 mg at week 0, 1 (subcutaneous)	106	NA	
	Control	76		
Elewski et al. (2022) [28]	Brodalumab 210 mg every 2 weeks (subcutaneous)	222		
	Control	219		
Gold et al. (2022) [26]	Apremilast 30 mg twice daily (oral)	297	49.2	58.6
	Control	298	48.3	50.7
van Voorhees et al. (2020) [23]	Apremilast 30 mg twice daily (oral)	201	47	62.2
	Control	102	46.5	60.8
Bagel et al. (2017) [30]	Guselkumab 100 mg at week 0, 4, 12 (subcutaneous)	51	42.7	41.2
	Control	51	41.1	52.9
Rich et al. (2016) [24] (ESTEEM 1)	Apremilast 30 mg twice daily (oral)	363	45.9	73.3
	Control	195	46.2	75.9
Rich et al. (2016) [24] (ESTEEM 2)	Apremilast 30 mg twice daily (oral)	175	46.2	70.9
	Control	91	45.7	84.6
Langley et al. (2015) [16]	Ixekizumab 10 mg at week 0, 2, 4, 8, 12, 16 (subcutaneous)	13	47.65	58
	Ixekizumab 25 mg at week 0, 2, 4, 8, 12, 16 (subcutaneous)	10	45.93	60
	Ixekizumab 75 mg at week 0, 2, 4, 8, 12, 16 (subcutaneous)	10	46.37	66
	Ixekizumab 150 mg at week 0, 2, 4, 8, 12, 16 (subcutaneous)	10	45.97	50
	Control	15	45	52

**FIGURE 2:**
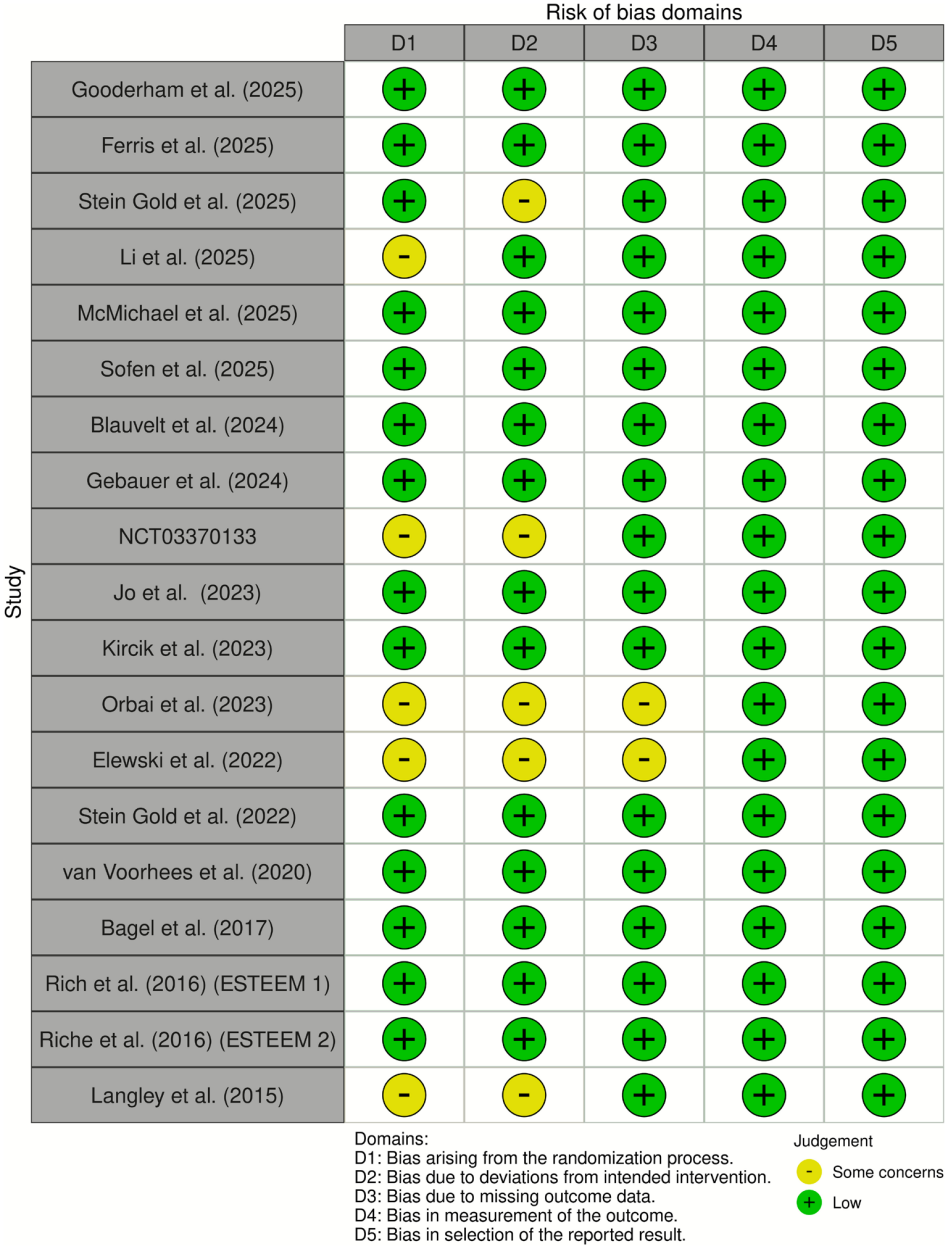
Evaluation of eligible studies' risk of bias (RoB). This is a pictorial summary of each eligible study's evidence quality using the Cochrane Collaboration's traffic plot.

Figure 1 summarizes the search process involved in identifying the eligible studies, and Table 1 presents a summary of the eligible trials’ study characteristics—while Table 2 lists the Food and Drug Administration (FDA) approval status of the active comparators. The “traffic plot” in Figure 2 depicts a qualitative summary of each eligible study's RoB. Results of node‐splitting analysis are in Table S1, and all network plots (for all 9 outcomes) are presented in the Appendix of the Supporting Information. Node‐splitting analysis was only feasible for the network corresponding to Sc‐PGA 0/1 at 16 weeks, where node‐splitting analysis showed no statistical evidence of inconsistency (*p* ≥ 0.05).

**TABLE 2:** FDA approval status of the active comparators.

Drug	Category	Indication	Initial FDA approval
Adalimumab (subcutaneous)	Biologic (TNF‐α inhibitor)	Chronic moderate‐to‐severe plaque psoriasis (adult)	2002
Apremilast (oral)	Oral small‐molecule (PDE‐4 inhibitor)	Plaque psoriasis (adults; pediatrics ≥ 6 years with weight‐based dosing)	2014
Bimekizumab (subcutaneous)	Biologic (IL‐17A/F inhibitor)	Moderate‐to‐severe plaque psoriasis (adult)	2023
Brodalumab (subcutaneous)	Biologic (IL‐17RA antagonist)	Adults with moderate‐to‐severe plaque psoriasis (with REMS)	2017
Deucravacitinib (oral)	Oral small‐molecule (TYK2 inhibitor)	Adults with moderate‐to‐severe plaque psoriasis	2022
Guselkumab (subcutaneous)	Biologic (IL‐23 p19 inhibitor)	Moderate‐to‐severe plaque psoriasis (adult)	2017
Icotrokinra (oral)	Oral peptide (IL‐23 receptor inhibitor)	Adults and adolescents ≥ 12 years with moderate‐to‐severe plaque psoriasis	Not FDA‐approved yet
Ixekizumab (subcutaneous)	Biologic (IL‐17A inhibitor)	Plaque psoriasis (adults; pediatrics ≥ 6 years)	2016
Roflumilast foam 0.3%	Topical non‐biologic (PDE‐4 inhibitor)	Plaque psoriasis of the scalp & body (≥ 12 years)	2025
Secukinumab (subcutaneous)	Biologic (IL‐17A inhibitor)	Moderate‐to‐severe plaque psoriasis (adults; pediatrics ≥ 6 years)	2015
Tildrakizumab (subcutaneous)	Biologic (IL‐23 p19 inhibitor)	Moderate‐to‐severe plaque psoriasis (adult)	2018

Abbreviations: IL‐17A inhibitor, interleukin‐17A inhibitor; IL‐17A/F inhibitor, interleukin‐17A/17F inhibitor; IL‐17RA antagonist, interleukin‐17 receptor A antagonist; IL‐23 p19 inhibitor, interleukin‐23 (p19 subunit) inhibitor; IL‐23 receptor inhibitor, interleukin‐23 receptor inhibitor; PDE‐4 inhibitor, phosphodiesterase‐4 inhibitor; REMS, risk evaluation and mitigation strategy; TYK2 inhibitor, tyrosine kinase 2 inhibitor.

**FIGURE 3:**
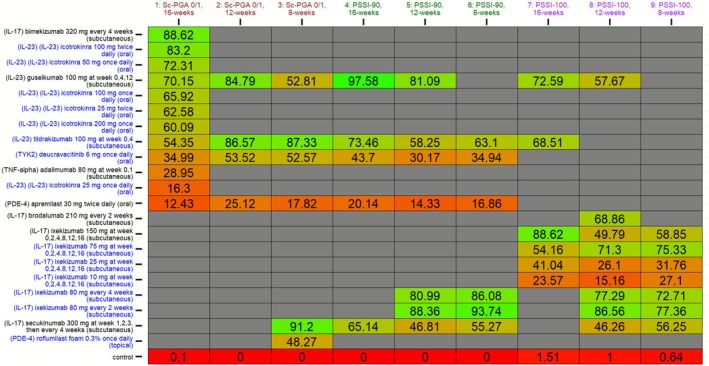
A kilim plot of comparators' surface under the cumulative ranking curve (SUCRA) values. Herein, the relative efficacy of each comparator, for each of the 9 networks, is presented in this kilim plot. The numeric values in cells correspond to comparators' SUCRA values. The horizontal axis corresponds to the outcome, and the vertical axis represents the distinct nodes (i.e., the comparators). A kilim plot aims to intuitively make comparators' efficacy visually comparable by use of a color gradient. For this kilim plot, greener cells correspond to higher efficacy, and redder cells correspond to lower efficacy. On the horizontal axis, each outcome (i.e., network) is suffixed by the total number of comparators therein in parentheses. Mg, milligrams; PSSI‐90, proportion of participants attaining 90% improvement in the Psoriasis Scalp Severity Index (at respective timepoint); PSSI‐100, proportion of participants attaining 100% improvement in the Psoriasis Scalp Severity Index (at respective timepoint); Sc‐PGA 0/1, proportion of participants achieving a scalp‐specific Physician Global Assessment of clear or almost clear (at the respective time point).

The “kilim plot” in Figure 3 presents the 21 active comparators’ SUCRA values across the 9 NMAs. Results of the base and sensitivity analyses were highly congruent; in other words, comparators’ rank orders were—by and large—highly similar even after (ecologically) adjusting for variation in age and sex. The SUCRA values from sensitivity analyses are presented in the kilim plot in Figure S1. The congruence in SUCRA values across the base and sensitivity models arguably supports that the base models were robust.

### 3.1 PSSI‐100

**FIGURE 4:**
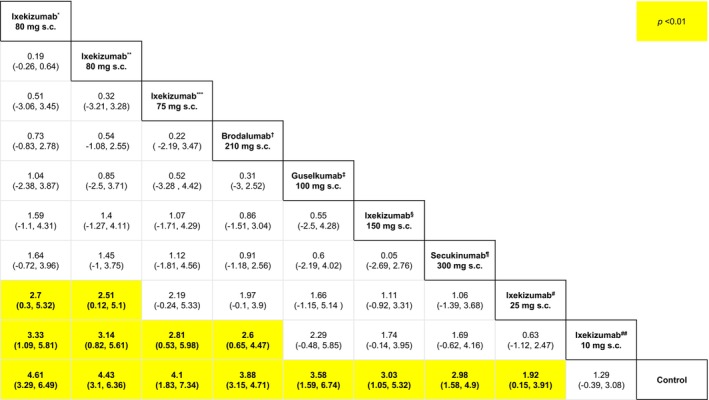
League table for PSSI‐100 at 12 weeks. This table presents relative efficacy for every pairwise comparison insofar as the outcome pertaining to the proportion of participants attaining 100% improvement in the Psoriasis Scalp Severity Index (PSSI‐100) at respective 12 weeks from baseline. s.c., subcutaneous injection. *Ixekizumab 80 mg every 2 weeks (subcutaneous), **Ixekizumab 80 mg every 4 weeks (subcutaneous), ***Ixekizumab 75 mg at weeks 0, 2, 4, 8, 12, 16 (subcutaneous), ^†^Brodalumab 210 mg every 2 weeks (subcutaneous), ^‡^Guselkumab 100 mg at weeks 0, 4, 12 (subcutaneous), ^§^Ixekizumab 150 mg at weeks 0, 2, 4, 8, 12, 16 (subcutaneous), ^¶^Secukinumab 300 mg at weeks 1, 2, 3 then every 4 weeks (subcutaneous), ^#^Ixekizumab 25 mg at weeks 0, 2, 4, 8, 12, 16 (subcutaneous), ^##^Ixekizumab 10 mg at weeks 0, 2, 4, 8, 12, 16 (subcutaneous).

Herein this paper, we present relative effects for the PSSI‐100 outcome measure with the largest number of comparators, that is, proportion of participants who attained PSSI‐100 at 12 weeks (10 comparators). We chose to present PSSI‐100 (over PSSI‐90) as it is arguably a more favorable outcome than PSSI‐90. Our NMA ranked ixekizumab 80 mg every 2 weeks (subcutaneous) to be the most efficacious comparator for PSSI‐100 at 12 weeks (SUCRA = 86.56%). The highest ranked option was not only significantly more efficacious than control (MD = 4.61, 95% CI: 3.29, 6.49, *p* < 0.05), but also significantly more efficacious than ixekizumab 10 mg at week 0, 2, 4, 8, 12, 16 (subcutaneous) (MD = 3.33, 95% CI: 1.09, 5.81, *p* < 0.05) and ixekizumab 25 mg at week 0, 2, 4, 8, 12, 16 (subcutaneous) (MD = 2.7, 95% CI: 0.3, 5.32, *p* < 0.05) (Figure 4).

We found that ixekizumab 150 mg at week 0, 2, 4, 8, 12, 16 (subcutaneous) was not significantly different from ixekizumab 80 mg every 2 weeks (subcutaneous) (*p* ≥ 0.05) (Figure 4). The only dosage of ixekizumab that brodalumab 210 mg every 2 weeks (subcutaneous) (SUCRA = 68.86%) was significantly greater than was the 10 mg dose, that is, ixekizumab 10 mg at week 0, 2, 4, 8, 12, 16 (subcutaneous) (SUCRA = 15.16%) (MD = 2.6, 95% CI: 0.65, 4.47, *p* < 0.05) (Figure 4).

### 3.2 Sc‐PGA

**FIGURE 5:**
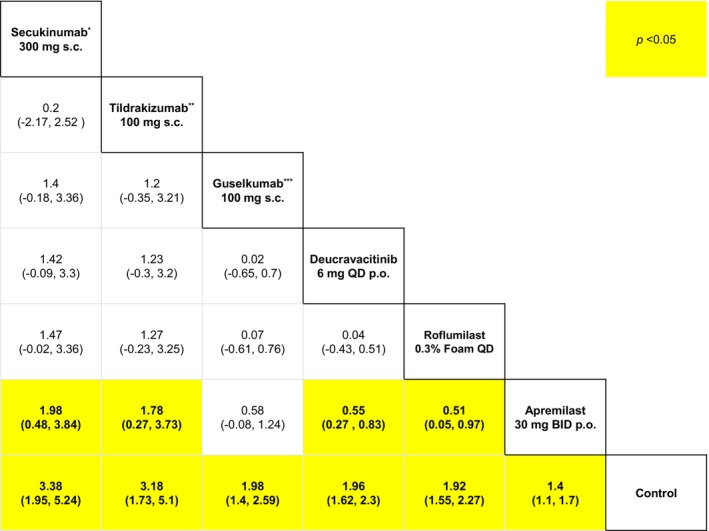
League table for Sc‐PGA 0/1 at 8 weeks. This table presents relative efficacy for every pairwise comparison of insofar as the outcome pertaining to the proportion of participants attaining scalp‐specific Physician Global Assessment of clear or almost clear (Sc‐PGA 0/1) at respective 8 weeks from baseline. BID, twice‐daily; p.o., per oral; QD, once‐daily; s.c., subcutaneous injection. *Secukinumab 300 mg at weeks 1, 2, 3, then every 4 weeks (subcutaneous), **Tildrakizumab 100 mg at weeks 0, 4 (subcutaneous), ***Guselkumab 100 mg at weeks 0, 4, 12 (subcutaneous).

**FIGURE 6:**
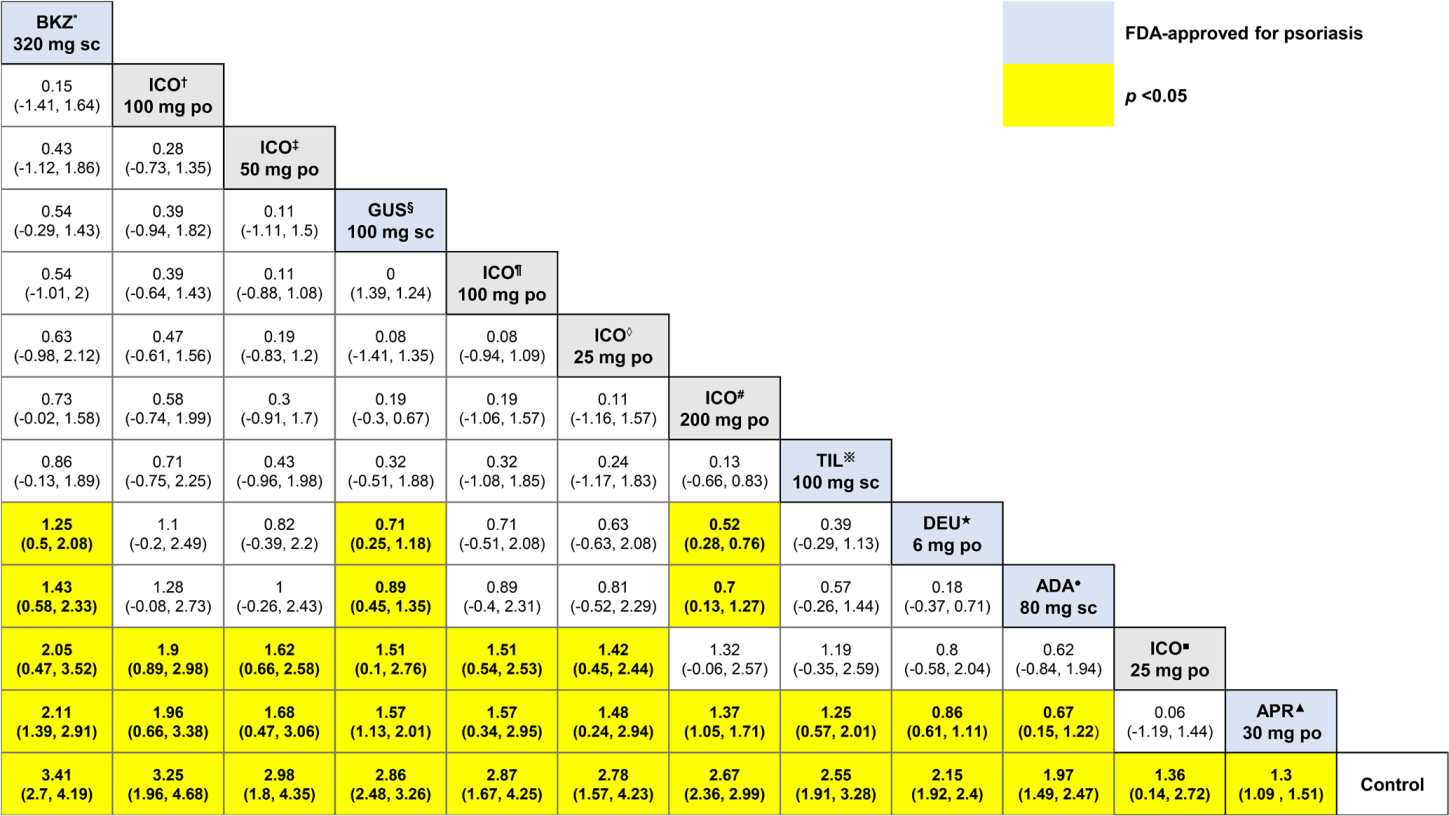
League table for Sc‐PGA 0/1 16 weeks. This table presents relative efficacy for every pairwise comparison insofar as the outcome pertaining to the proportion of participants attaining scalp‐specific Physician Global Assessment of clear or almost clear (Sc‐PGA 0/1) at respective 16 weeks from baseline. po, per oral; s.c., subcutaneous injection. *(IL‐17) bimekizumab 320 mg every 4 weeks (subcutaneous), ^†^(IL‐23) icotrokinra 100 mg twice daily (oral), ^‡^(IL‐23) icotrokinra 50 mg once daily (oral), ^§^(IL‐23) guselkumab 100 mg at weeks 0, 4, 12 (subcutaneous), ^¶^(IL‐23) icotrokinra 100 mg once daily (oral), ◊(IL‐23) icotrokinra 25 mg twice daily (oral), ^#^(IL‐23) icotrokinra 200 mg once daily (oral), ※(IL‐23) tildrakizumab 100 mg at weeks 0, 4 (subcutaneous), ★(TYK2) deucravacitinib 6 mg once daily (oral), ●(TNF‐α) adalimumab 80 mg at weeks 0, 1 (subcutaneous), ■ (IL‐23) icotrokinra 25 mg once daily (oral), ▲(PDE‐4) apremilast 30 mg twice daily (oral).

Herein, we present the relative effects for the Sc‐PGA 0/1 outcomes corresponding to the proportion of participants who attained Sc‐PGA of 0 or 1 at 8 weeks (Figure 5) and 16 weeks (Figure 6).

At 8 weeks, secukinumab 300 mg at week 1, 2, 3, then every 4 weeks (subcutaneous) was the most efficacious option (SUCRA = 91.2%). Our analysis found that secukinumab 300 mg at week 1, 2, 3, then every 4 weeks (subcutaneous) was significantly more efficacious than apremilast 30 mg twice daily (oral) (SUCRA = 17.82%) (MD = 1.98, 95% CI: 0.48, 3.84, *p* < 0.05) (Figure 5). Our analysis also found that deucravacitinib 6 mg once daily (oral) (SUCRA = 52.57%) was significantly more efficacious than apremilast 30 mg twice daily (oral) (MD = 0.55, 95% CI: 0.27, 0.83, *p* < 0.05) (Figure 5). We also found that roflumilast foam 0.3% once daily (topical) (SUCRA = 48.27%) was significantly more efficacious than apremilast 30 mg twice daily (oral) (MD = 0.51, 95% CI: 0.05, 0.97, *p* < 0.05) (Figure 5). Our results showed that tildrakizumab 100 mg at week 0, 4 (subcutaneous) (SUCRA = 87.83%) was significantly more efficacious than apremilast 30 mg twice daily (oral) (MD = 1.78, 95% CI: 0.27, 3.73, *p* < 0.05) (Figure 5).

At 16 weeks, bimekizumab 320 mg every 4 weeks (subcutaneous) was the most efficacious option (SUCRA = 88.62%) (Figure 6); this highest‐ranked option was significantly more efficacious than deucravacitinib 6 mg once daily (oral) (MD = 1.25, 95% CI: 0.5, 2.08, *p* < 0.05), apremilast 30 mg twice daily (oral) (MD = 2.11, 95% CI: 1.39, 2.91, *p* < 0.05), and adalimumab 80 mg at week 0, 1 (subcutaneous) (MD = 1.43, 95% CI: 0.58, 2.33, *p* < 0.05) (Figure 6). While bimekizumab 320 mg every 4 weeks (subcutaneous) was significantly more efficacious than the lower dosage regimens of icotrokinra, it was not significantly more efficacious than the higher dosage regimens of the IL‐23 inhibitor (Figure 6). In fact, the only dosage regimen of icotrokinra that bimekizumab 320 mg every 4 weeks (subcutaneous) was significantly more efficacious than was the icotrokinra 25 mg once daily (oral) regimen (i.e., the lowest of the dosages) (MD = 2.05, 95% CI: 0.47, 3.52, *p* < 0.05).

## 4 Discussion

Using published randomized evidence within the past decade, the current study has determined the relative efficacy of monotherapy with immunomodulatory agents on scalp psoriasis. We have compared the therapeutic impact of various agents, namely, adalimumab, apremilast, deucravacitinib, bimekizumab, secukinumab, ixekizumab, brodalumab, guselkumab, tildrakizumab, icotrikinra, and ixekizumab; the nodes of our networks were at the level of dosage; our search identified various dosage regimens of ixekizumab.

Bimekizumab, secukinumab, brodalumab, and ixekizumab are inhibitors of interleukin (IL)‐17 receptors [33, 34, 35]; guselkumab, tildrakizumab, and icotrokinra are inhibitors of IL‐23 receptors [36] deucravacitinib is a tyrosine kinase 2 (TYK2) inhibitor [33].

Though Kaur and colleagues published their work in 2025, their search strategy covered studies till October 2022 [4]. Hence, our search was able to capture studies that have investigated agents whose relative efficacy has not been determined previously: the current study is the first to produce comparative evidence for the relative efficacy of icotrokinra, tildrakizumab, roflumilast, and deucravacitinib in an NMA study. Though Kaur et al.'s NMA study compared the efficacy of ixekizumab, our work compared dosages of ixekizumab that the authors could not: our NMA study is the first to provide comparative evidence on the 25, 75, 10, 80 mg doses of ixekizumab, for example.

Our findings are congruent with Kaur et al.'s findings. For instance, Kaur et al. also found bimekizumab 320 mg every 4 weeks (subcutaneous) to be the most efficacious monotherapy for Sc‐PGA 0/1.

Our work has some limitations. A sensitivity analysis accounting for variation in baseline disease severity was not performed as many of the eligible studies did not provide mean Psoriasis Scalp Severity Index (PSSI) at baseline. Moreover, we deemed the baseline Psoriasis Area Severity Index (PASI) to not be a suitable proxy for mean baseline PSSI, as one is not necessarily a barometer for the other; for example, severe plaque psoriasis can exist in the absence of scalp psoriasis. Additionally, this study only analyzed the adult population: we did not evaluate the treatments' relative efficacy in the population aged below 18 years. Furthermore, given that insurance, drug acquisition costs, rebates, and dosing assumptions vary by jurisdiction, we did not conduct a de novo pharmacoeconomic analysis. Instead, we evaluated comparative effectiveness based primarily on clinical outcomes.

Until recently and before the advent of monoclonal antibodies and janus kinase (JAK) inhibitors, there were relatively fewer therapeutic options for treating severe scalp psoriasis that was unresponsive or poorly responsive to topical corticosteroids and calcipotriene, and the “traditional” agents (methotrexate, acitretin, and cyclosporine) [34]. Our analyses excluded these agents and phototherapy.

Results from our NMA would guide the use of the recently proliferated treatment options for scalp psoriasis. A strength of our work is that we did not merge various time points into one node. Also, for any given therapeutic agent, we did not merge the different dosage regimens; for example, our nodes distinguished between ixekizumab 80 mg every 2 weeks (subcutaneous) and ixekizumab 80 mg every 4 weeks (subcutaneous). At the agent level, we compared the following using established outcome measures (i.e., Physician Global Assessment for scalp and Psoriasis Scalp Severity Index), namely, icotrekinra, deucravacitinib, tildrakizumab, and roflumilast (not previously compared), and adalimumab, apremilast, bimekizumab, brodalumab, guselkumab, ixekizumab, and secukinumab. According to Wechter et al., PSSI is the most commonly used physician‐reported measure for scalp psoriasis; the ScPGA is frequently used in many clinical trials [35].

The current study did not compare cyclosporine (a calcineurin inhibitor), methotrexate (an antineoplastic agent), and acitretin (a retinoid) because we did not find randomized studies that investigated their efficacy using the aforementioned outcome measures for scalp psoriasis. Nonetheless, these agents are frequently used in clinical practice to treat plaque psoriasis and—by extension—psoriasis of the scalp.

As per our analyses, we observed that an IL‐17 inhibitor was ranked most efficacious—vis‐à‐vis the options of IL‐23 inhibitors. In light of expert opinion, IL‐23 inhibitors may be more efficacious, more so on a long‐term basis. Hence, as more data accumulates in the evidence base, future studies may need to investigate these agents' efficacy insofar as long‐term effects. With the exception of bimekizumab 320 mg every 4 weeks, the efficacy of the IL‐23 inhibitors was generally higher than that of IL‐17 inhibitors.

Notably, evidence on whether sex/gender modifies systemic treatment response in psoriasis is mixed across real‐world cohorts and evidence syntheses. In a large two‐country registry analysis (PsoBest/SDNTT), women had slightly higher PASI response rates after 3, 6, and 12 months. Women treated with biologics achieved significantly higher response rates (PASI ≤ 3) than men at both the 3‐month (57.8% vs. 48.5%; *p* ≤ 0.004) and 6‐month intervals (69.2% vs. 60.9%; *p* ≤ 0.018). In contrast, other observational datasets using drug survival as an effectiveness proxy have reported no meaningful sex differences in effectiveness. However, modest differences in adverse‐event rates may exist. Women reported slightly more adverse events compared to men [37, 38]. Our study is the first to provide comparative evidence on recent immunomodulatory agent's efficacy at specific timepoints—unlike Kaur et al.'s NMA study that amalgamated outcome data from 4 to 16 weeks.

## 5 Conclusion

This network meta‐analysis of randomized trials in scalp psoriasis compared newly evaluated agents with established outcome measures. At 16 weeks, ixekizumab 150 mg (given at weeks 0, 2, 4, 8, and 12) ranked highest for PSSI‐100, while bimekizumab 320 mg every 4 weeks led for Sc‐PGA 0/1. At 8 weeks, ixekizumab 80 mg every 2 weeks topped PSSI‐100, and secukinumab 300 mg (weeks 1–3, then every 4 weeks) ranked highest for Sc‐PGA 0/1. Small‐molecule therapies (apremilast, deucravacitinib, roflumilast) showed modest benefit. Most of the comparators are delivered subcutaneously with oral administration for deucravacitinib and apremilast. Roflumilast foam 0.3% is applied topically. These findings can inform clinical decision‐making and future trial design. Nevertheless, longer, head‐to‐head, psoriasis scalp‐specific studies with standardized outcomes are needed to confirm comparative effectiveness and durability.

## Author Contributions

Conception of the manuscript was done by A.K.G. and M.T. Data analysis was performed by M.A.B. The manuscript was drafted by A.K.G., T.W., V.P., M.T., and M.A.B, substantively edited, and revised by A.K.G., M.A.B., V.P., and M.T.

## Funding

The authors have nothing to report.

## Ethics Statement

The authors have nothing to report.

## Consent

The authors have nothing to report.

## Conflicts of Interest

The authors declare no conflicts of interest.

## Supporting information


**Data S1:** jocd70662‐sup‐0001‐Supinfo.pdf.

## Data Availability

The data that support the findings of this study are available from the corresponding author upon reasonable request.
