# Supplementary material for: Relative Efficacy of Immunomodulatory Monotherapies for Psoriasis of the Scalp: A Network Meta‐Analysis Study
Source: J Cosmet Dermatol. 2026 Jan 8;25(1):e70662. doi: 10.1111/jocd.70662 (PMC12780935; doi:10.1111/jocd.70662)
Supplement: Supplementary file 1 — Data S1: jocd70662‐sup‐0001‐Supinfo.pdf. [file JOCD-25-e70662-s001.pdf]

Title:

Relative efficacy of immunomodulatory monotherapies for psoriasis of the scalp: a network meta-analysis study

Supplement

Supplementary Table 1

Node-splitting analysis of inconsistency for the network pertaining to SC-PGA 0/1 at 16 weeks

apremilast 30 mg twice daily (oral) vs. deucravacitinib 6 mg once daily (oral)		
**Comparison**	**Credible interval**	**p-value**
Direct	1.1 (0.62, 1.5)	0.053825
Indirect	0.36 (-0.20, 0.92)	
network	0.80 (0.36, 1.1)	

Network plot for Sc−PGA at 8 weeks

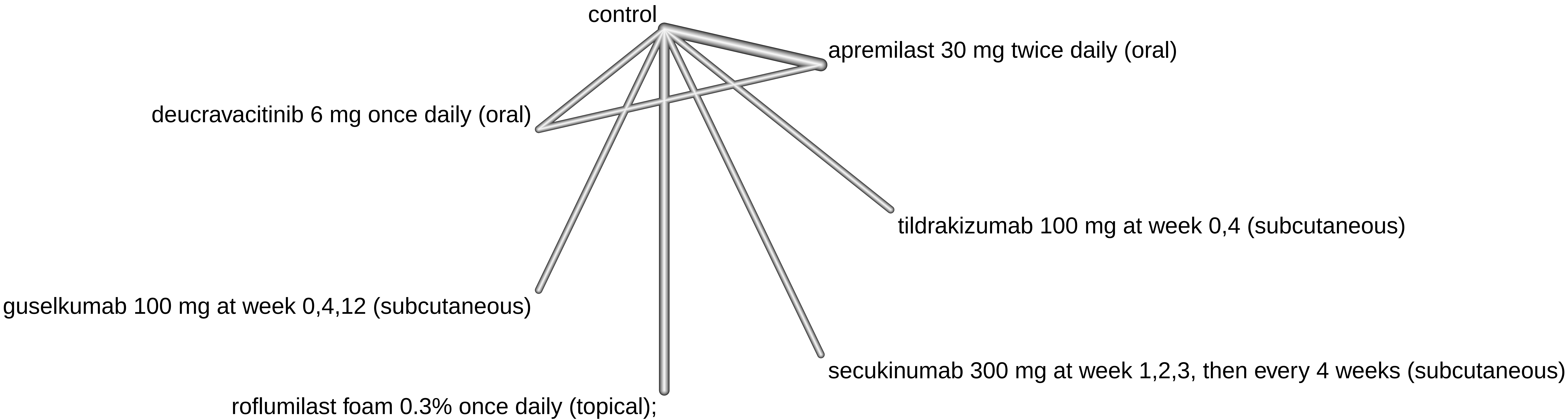

Network plot for Sc−PGA at 12 weeks

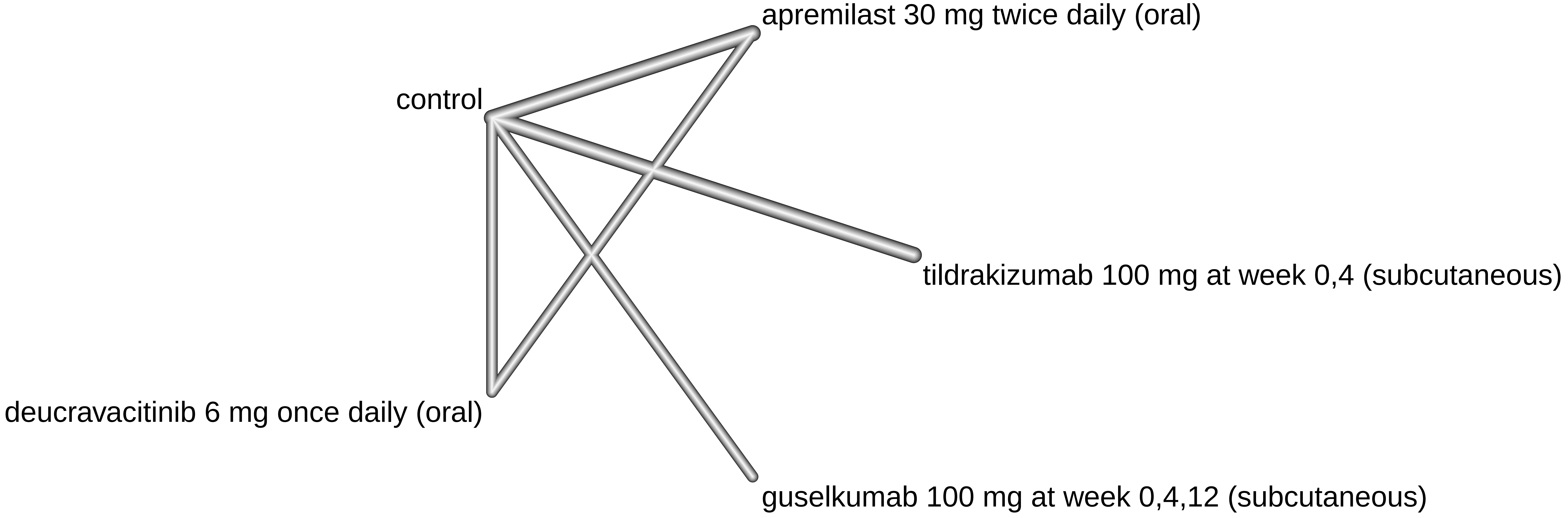

Network plot for Sc−PGA at 16 weeks

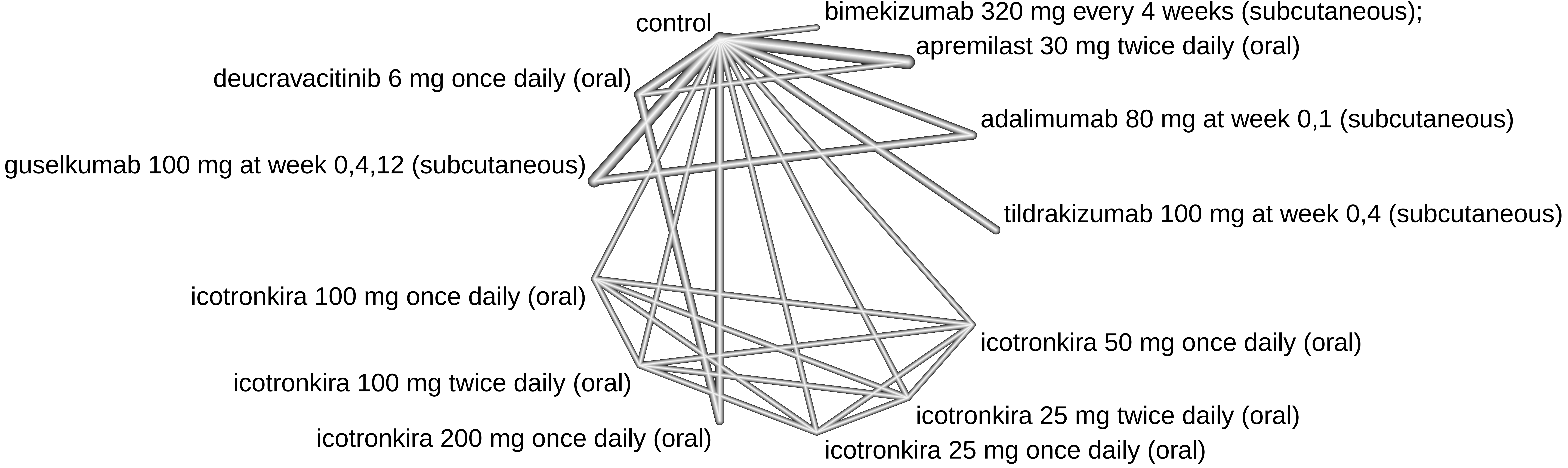

Network plot for PSSI−90 at 8 weeks

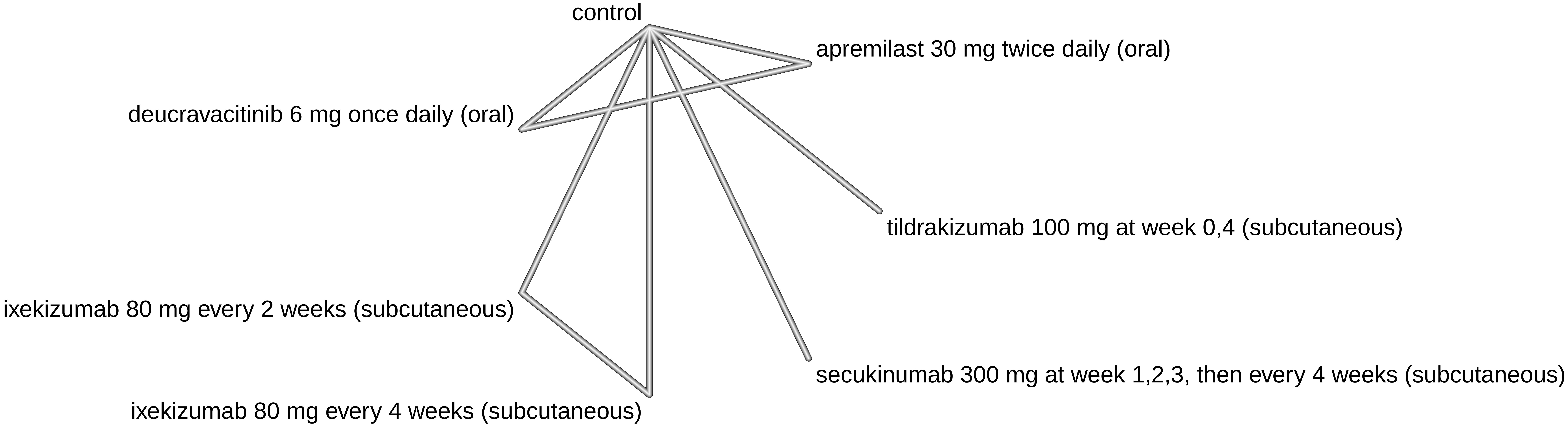

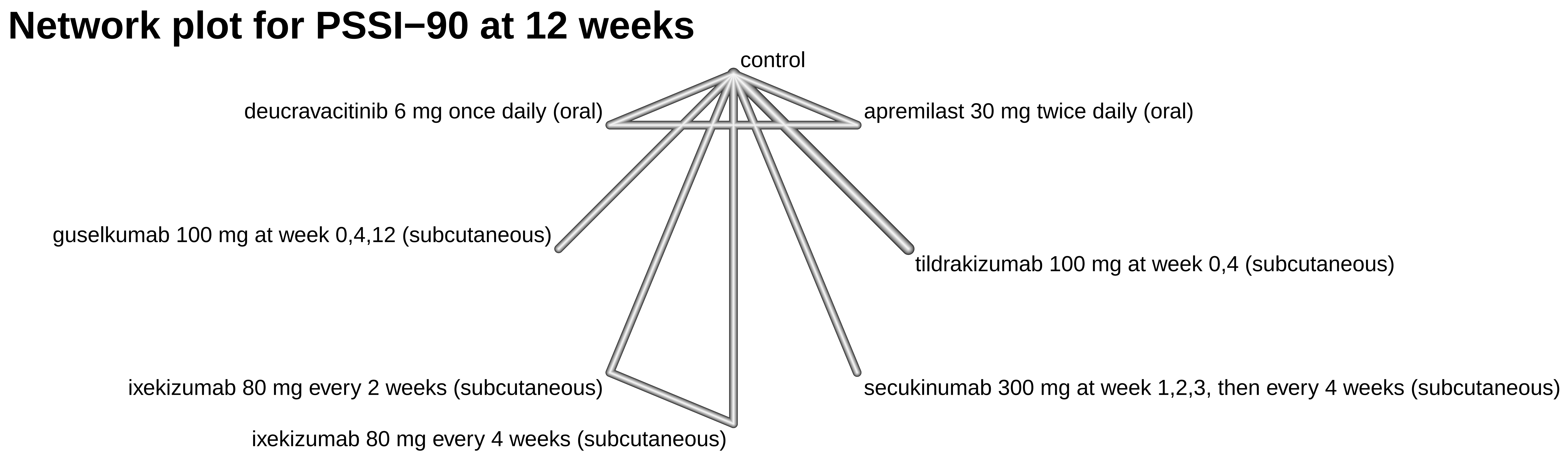
Network plot for PSSI−90 at 12 weeks
control
deucravacitinib 6 mg once daily (oral)
apremilast 30 mg twice daily (oral)
guselkumab 100 mg at week 0,4,12 (subcutaneous)
tildrakizumab 100 mg at week 0,4 (subcutaneous)
ixekizumab 80 mg every 2 weeks (subcutaneous)
secukinumab 300 mg at week 1,2,3, then every 4 weeks (subcutaneous)
ixekizumab 80 mg every 4 weeks (subcutaneous)

Network plot for PSSI−90 at 16 weeks

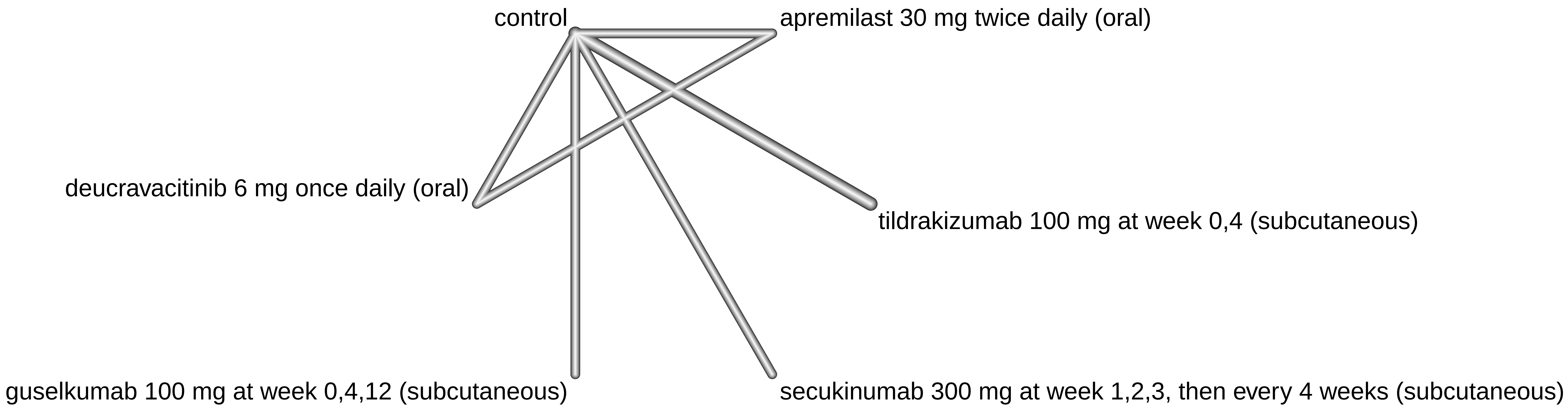

Network plot for PSSI−100 at 8 weeks

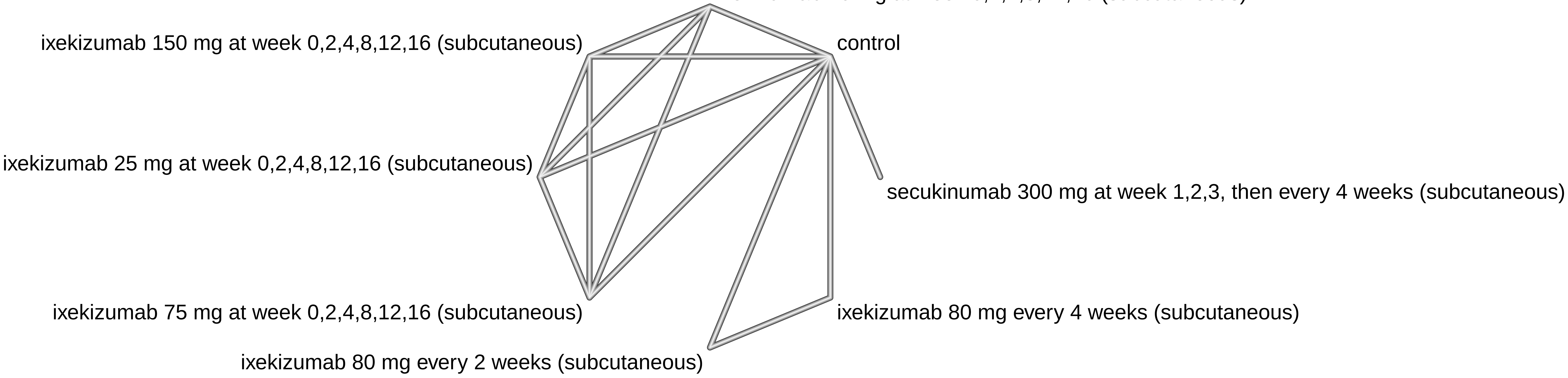

Network plot for PSSI−100 at 12 weeks

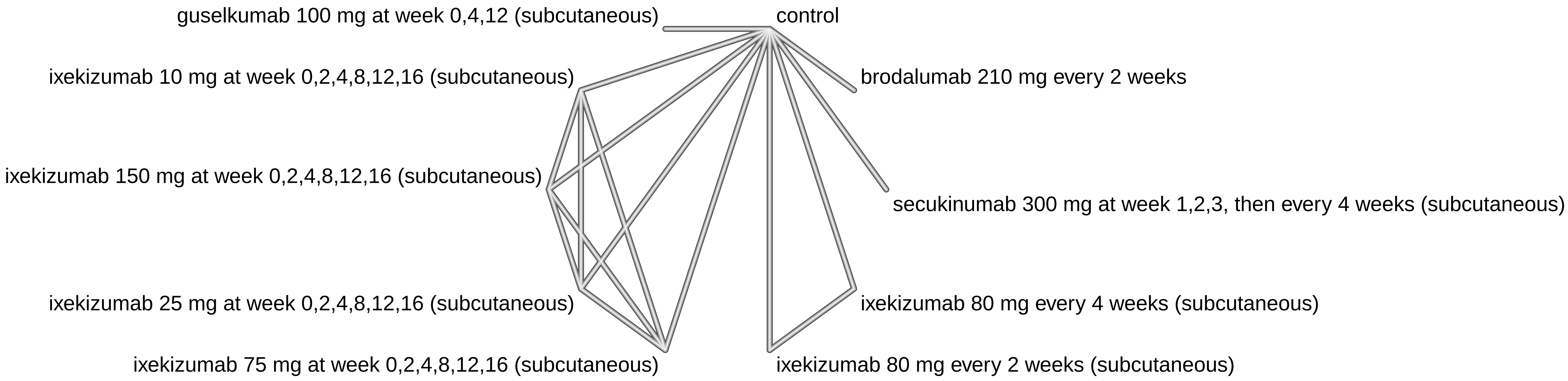

Network plot for PSSI−100 at 16 weeks

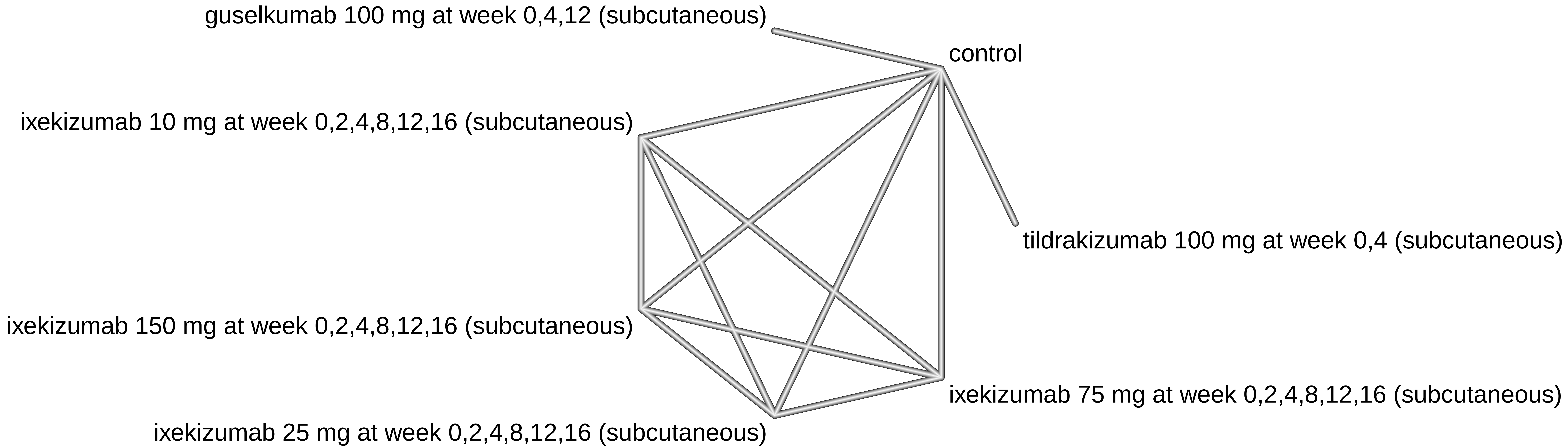

Supplementary Figure 1

	1: Sc-PGA 0/1 16-weeks	1a : Sc-PGA 0/1 16-weeks	1b : Sc-PGA 0/1 16-weeks	2: Sc-PGA 0/1 12-weeks	2a : Sc-PGA 0/1 12-weeks	2b : Sc-PGA 0/1 12-weeks	3: Sc-PGA 0/1 8-weeks	3a : Sc-PGA 0/1 8-weeks	3b : Sc-PGA 0/1 8-weeks	4: PSSI-90 16-weeks	4a : PSSI-90 16-weeks	4b : PSSI-90 16-weeks	5: PSSI-90 12-weeks	5a : PSSI-90 12-weeks	5b : PSSI-90 12-weeks	6: PSSI-90 8-weeks	6a : PSSI-90 8-weeks	6b : PSSI-90 8-weeks	7: PSSI-100 16-weeks	7a : PSSI-100 16-weeks	7b : PSSI-100 16-weeks	8: PSSI-100 12-weeks	8a : PSSI-100 12-weeks	8b : PSSI-100 12-weeks	9: PSSI-100 8-weeks	9a : PSSI-100 8-weeks	9b : PSSI-100 8-weeks
(IL-17) bimekizumab 320 mg every 4 weeks (subcutaneous)	88.62	95.83	96.86																								
(IL-23) (IL-23) icotrokinra 100 mg twice daily (oral)	83.2																										
(IL-23) (IL-23) icotrokinra 50 mg once daily (oral)	72.31																										
(IL-23) guselkumab 100 mg at week 0,4,12 (subcutaneous)	70.15	83.42	85.37	84.79	59.36	96.44	52.81	57.21	56.75	97.58	71.85	60.59	81.09	70.29	63.26				72.59	52.66	58.33	57.67	51.56	57.11			
(IL-23) (IL-23) icotrokinra 100 mg once daily (oral)	65.92																										
(IL-23) (IL-23) icotrokinra 25 mg twice daily (oral)	62.58																										
(IL-23) (IL-23) icotrokinra 200 mg once daily (oral)	60.09																										
(IL-23) tildrakizumab 100 mg at week 0,4 (subcutaneous)	54.35	62.96	52.51	86.57	91.66	57.29	87.33	84.16	77.9	73.46	68.95	69.72	58.25	58.94	56.73	63.1	57.6	42.18	68.51	72.66	60.32						
(TYK2) deucravacitinib 6 mg once daily (oral)	34.99	51.02	59.58	53.52	69.54	64.23	52.57	45.6	55.37	43.7	51.46	62.34	30.17	34.73	43.31	34.94	25.78	44.83									
(TNF-alpha) adalimumab 80 mg at week 0,1 (subcutaneous)	28.95	39.26	33.53																								
(IL-23) (IL-23) icotrokinra 25 mg once daily (oral)	16.3																										
(PDE-4) apremilast 30 mg twice daily (oral)	12.43	17.51	22.15	25.12	29.4	32.01	17.82	18.08	21.15	20.14	34.78	31.82	14.33	23.77	25.96	16.86	25.27	42									
(IL-17) brodalumab 210 mg every 2 weeks (subcutaneous)																						68.86					
(IL-17) ixekizumab 150 mg at week 0,2,4,8,12,16 (subcutaneous)																			88.62	78.87	72.5	49.79	55.85	53.79	58.85	58.11	64.23
(IL-17) ixekizumab 75 mg at week 0,2,4,8,12,16 (subcutaneous)																			54.16	45.08	49.3	71.3	65.87	60.76	75.33	63.78	45.24
(IL-17) ixekizumab 25 mg at week 0,2,4,8,12,16 (subcutaneous)																			41.04	44.25	47.41	26.1	34.53	37.24	31.76	37.34	33.71
(IL-17) ixekizumab 10 mg at week 0,2,4,8,12,16 (subcutaneous)																			23.57	23.77	36.98	15.16	20.65	28.29	27.1	30.63	35.75
(IL-17) ixekizumab 80 mg every 4 weeks (subcutaneous)													80.99	72.57	73.43	86.08	79.41	75.16				77.29	75.33	71.69	72.71	69.46	68.31
(IL-17) ixekizumab 80 mg every 2 weeks (subcutaneous)													88.36	72.16	72.86	93.74	68.54	65.18				86.56	75.52	72.45	77.36	69.47	61.49
(IL-17) secukinumab 300 mg at week 1,2,3, then every 4 weeks (subcutaneous)							91.2	91.89	96.17	65.14	47.84	47.36	46.81	48.22	47.77	55.27	65.72	64.1				46.26	45.69	52.41	56.25	49.39	67.24
(PDE-4) roflumilast foam 0.3% once daily (topical)							48.27	53.05	42.65																		
control	0.1	0	0	0	0.04	0.03	0	0	0.01	0	25.12	28.16	0	19.33	16.68	0	27.68	16.55	1.51	32.7	25.17	1	24.99	16.26	0.64	21.81	24.04
